# The Soft Catheter in Plugging the Nose

**Published:** 1898-09

**Authors:** 


### THE SOFT CATHETER IN PLUGGING THE NOSE.

Dr. M. P. Smithwick, in a recent issue of the *Boston Medical and Surgical Journal*, says:

Employment of the soft rubber catheter in plugging the nose requires no suggestion to those familiar with its use in nasal feeding. Except in nasal operations under a general anæsthetic, it is rarely necessary to plug the nose. Experts are usually correct in considering the frequency with which one resorts to this procedure in cases of spontaneous epistaxis to be inversely as his skill. A soft rubber catheter, a string more than twice the catheter's length (waxed dental floss is excellent), a wire (the stilletto of a gum-elastic catheter will do if the soft catheter is shortened), material for a plug, some styptic and a lubricant are necessary. A solution of cocaine is helpful and may be necessary to shrink the parts. Forceps are convenient. An end of the wire is passed through the catheter. Bring the ends of string together behind the catheter. Lubricate catheter; pass along floor of nostril, and, when the tip appears below the soft palate, grasp with forceps and draw it out of the mouth. Draw out the half of string not contained in the catheter. An end of the catheter carrying an end of string now projects from a nostril and from the mouth. Attach the plug and draw it near to the catheter's tip, leaving outside the plug sufficient string to project from the mouth when the plug is in position. Then withdraw from the nose the catheter and, until the plug is fixed firmly in position, the string simultaneously. Considerable swelling, at least of the soft palate, will follow. If the plug is aseptic there need be no apprehension.—*Charlotte Medical Journal.*

